# The genome sequence of the ornate tailed digger wasp, *Cerceris rybyensis* (Linnaeus, 1771)

**DOI:** 10.12688/wellcomeopenres.17483.1

**Published:** 2021-12-13

**Authors:** Liam Crowley, University of Oxford and Wytham Woods Genome Acquisition Lab, Darwin Tree of Life Barcoding collective, Wellcome Sanger Institute Tree of Life programme, Wellcome Sanger Institute Scientific Operations: DNA Pipelines collective, Tree of Life Core Informatics collective, Darwin Tree of Life Consortium

**Affiliations:** 1 Department of Zoology, University of Oxford, Oxford, UK

**Keywords:** Cerceris rybyensis, ornate tailed digger wasp, genome sequence, chromosomal, Hymenoptera

## Abstract

We present a genome assembly from an individual female *Cerceris rybyensis *(the ornate tailed digger wasp; Arthropoda; Insecta; Lepidoptera; Noctuidae). The genome sequence is 574 megabases in span. The majority of the assembly, 89.81%, is scaffolded into 14 chromosomal pseudomolecules.

## Species taxonomy

Eukaryota; Metazoa; Ecdysozoa; Arthropoda; Hexapoda; Insecta; Pterygota; Neoptera; Endopterygota; Hymenoptera; Apocrita; Aculeata; Apoidea; Spheciformes; Crabronidae; Philanthinae; Cercerini; Cerceris; *Cerceris rybyensis* (Linnaeus, 1771) (NCBI:txid1167272).

## Background

The Ornate Tailed Digger Wasp *, Cerceris rybyensis,* is a widespread, mid-sized (6–12 mm) digger wasp that occurs throughout the Palearctic. In the UK it is common across southern England in habitats with sandy soil and on chalk grassland. Adults are yellow and black with a distinctively ribbed metasoma characteristic of the Genus, that is irregularly banded black and yellow in this species, distinguishing it from other *Cerceris* species in the UK.

It is a univoltine species, with a flight period from late June to early September. It often nests in dense aggregations, occasionally intermixed with *Cerceris arenaria.* Adult females dig a 10–15 cm deep burrow with multiple cells branching off laterally ( Else, 1997). The females hunt small to medium sized bees of several genera, particularly *Lasioglossum* and *Halictus.* Often prey returning to their nest fully laden with pollen are favoured, although males and unladen females are also taken. Navigation back to the nest is aided by the undertaking of arcing orientation flights around the nest as they leave ( Zeil, 1993). Prey is paralysed by stinging and malaxated just behind the head, before being carried back to a pre-prepared nest cell within the burrow. Paralysis is achieved by the delivery of venom via the stinger, which contains a complex mix of biogenic amines, peptides and proteins that act as toxins, neuromodulators, immunomodulators, metabolic-modulators and antimicrobial agents ( Kote *et al.*, 2019). Each cell is stocked with 5–8 paralysed bees, which remain alive for around 2 days ( Lomholdt, 1975). A single egg is laid in each cell, which hatches and consumes the prey provisions before pupating. Individuals remain within the pupal cocoon over winter. Adults visit the flowers of various species to feed on nectar, including common hogweed ( *Heracleum sphondylium*), wild carrot ( *Daucus carota*), yarrow ( *Achillea millefolium*) and creeping thistle ( *Cirsium arvense*).

## Genome sequence report

**Figure 1.**
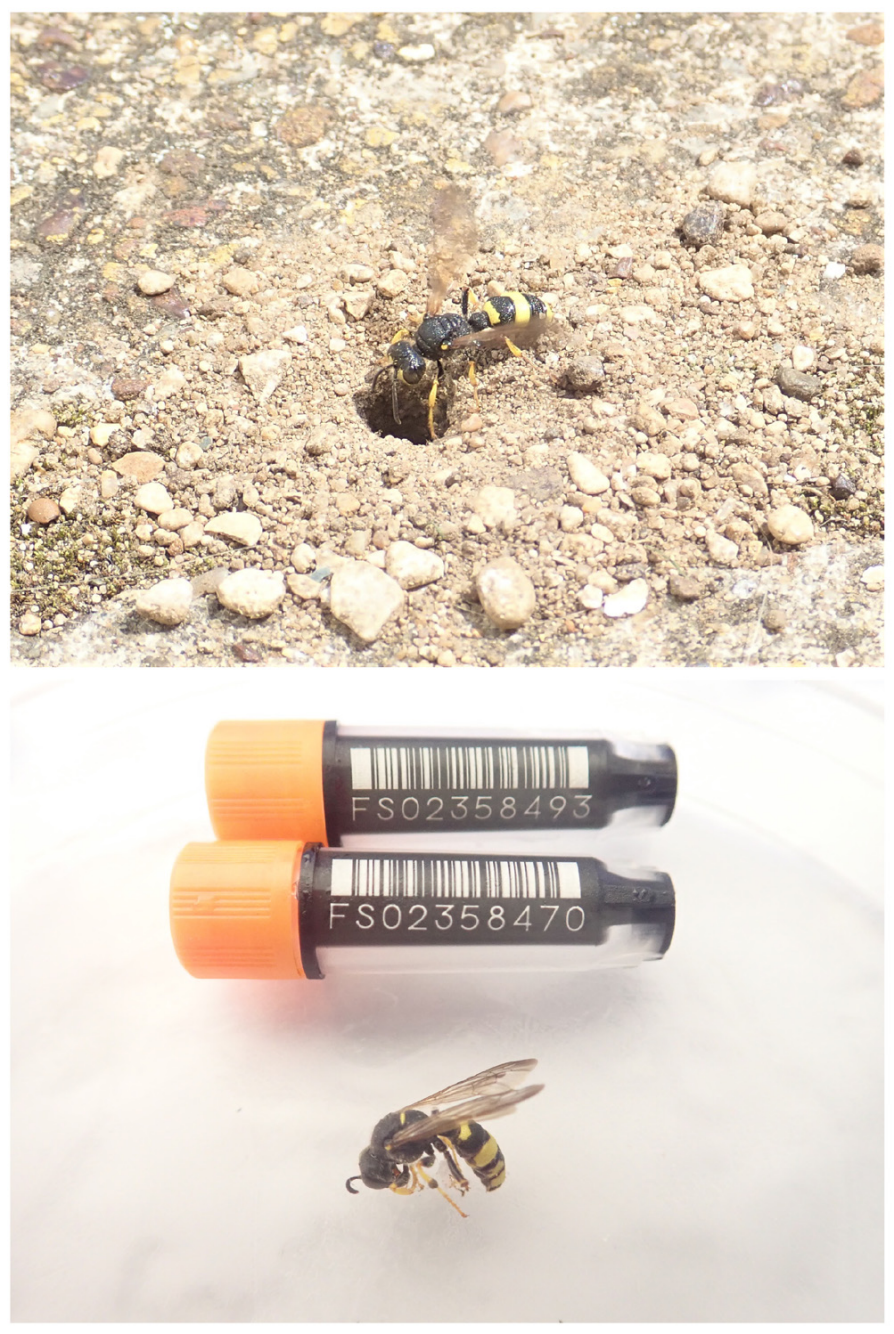
Image of the iyCerRyby1 specimen taken prior to capture (top) and during preservation and processing (bottom).

The genome was sequenced from a single female *C. rybyensis* ( Figure 1) collected from Wytham Woods, Oxfordshire, UK (latitude 51.782, longitude -1.316). A total of 58-fold coverage in Pacific Biosciences single-molecule, circular consensus (HiFi) long reads and 91-fold coverage in 10X Genomics read clouds were generated. Primary assembly contigs were scaffolded with chromosome conformation Hi-C data. Manual assembly curation corrected 13 missing/misjoins and removed 1 haplotypic duplication, reducing the scaffold number by 1.47%.

**Figure 2.**
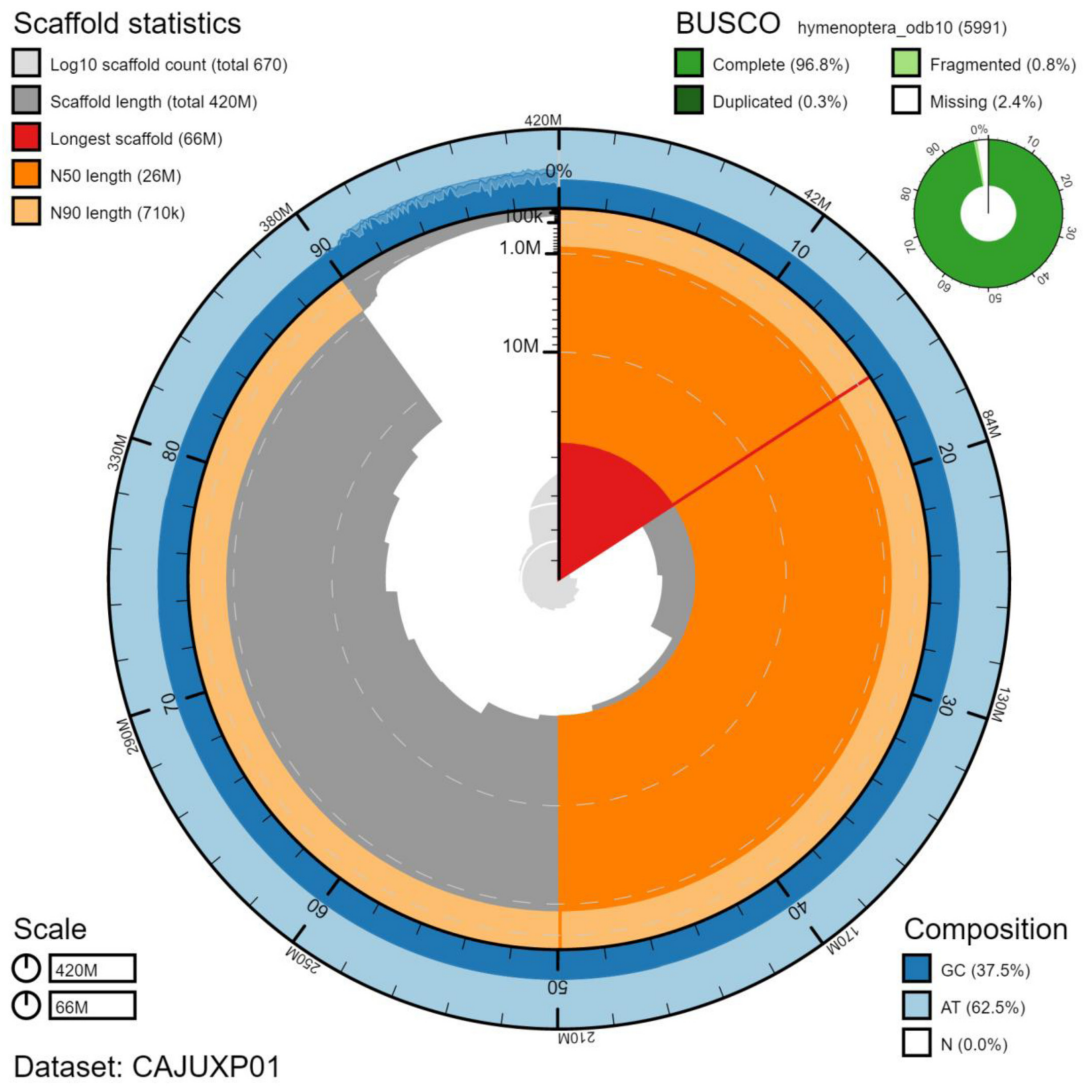
Genome assembly of *Cerceris rybyensis*, iyCerRyby1.1: metrics. The BlobToolKit Snailplot shows N50 metrics and BUSCO gene completeness. The main plot is divided into 1,000 size-ordered bins around the circumference with each bin representing 0.1% of the 418,093,278 bp assembly. The distribution of chromosome lengths is shown in dark grey with the plot radius scaled to the longest chromosome present in the assembly (66,377,818 bp, shown in red). Orange and pale-orange arcs show the N50 and N90 chromosome lengths (26,175,038 and 705,187 bp), respectively. The pale grey spiral shows the cumulative chromosome count on a log scale with white scale lines showing successive orders of magnitude. The blue and pale-blue area around the outside of the plot shows the distribution of GC, AT and N percentages in the same bins as the inner plot. A summary of complete, fragmented, duplicated and missing BUSCO genes in the hymenoptera_odb10 set is shown in the top right. An interactive version of this figure is available at https://blobtoolkit.genomehubs.org/view/iyCerRyby1.1/dataset/CAJUXP01/snail.

The final assembly has a total length of 574 Mb in 51 sequence scaffolds with a scaffold N50 of 19.0 Mb ( Table 1). Of the assembly sequence, 89.81% was assigned to 14 chromosomal-level scaffolds, representing 14 autosomes (numbered by sequence length) ( Figure 2– Figure 5; Table 2). A large number of repetitive scaffolds were produced in sequencing, which were not able to be placed in the assembly. The assembly has a BUSCO v5.1.2 ( Manni *et al.*, 2021) completeness of 96.8% (single 96.6%, duplicated 0.3%) using the hymenoptera_odb10 reference set. While not fully phased, the assembly deposited is of one haplotype. Contigs corresponding to the second haplotype have also been deposited.

**Table 1.** Genome data for *Cerceris rybyensis*, iyCerRyby1.1.

*Project accession data*	
Assembly identifier	iyCerRyby1.1
Species	*Cerceris rybyensis*
Specimen	iyCerRyby1
NCBI taxonomy ID	NCBI:txid1167272
BioProject	PRJEB45199
BioSample ID	SAMEA7701329
Isolate information	Female, abdomen (genome assembly), head/thorax (Hi-C)
*Raw data accessions*	
PacificBiosciences SEQUEL II	ERR6436387
10X Genomics Illumina	ERR6054972-ERR6054975
Hi-C Illumina	ERR6054976
*Genome assembly*	
Assembly accession	GCA_910591515.1
*Accession of alternate haplotype*	GCA_910591445.1
Span (Mb)	418
Number of contigs	710
Contig N50 length (Mb)	15.2
Number of scaffolds	671
Scaffold N50 length (Mb)	26.2
Longest scaffold (Mb)	66.4
BUSCO * genome score	C:96.8%[S:96.6%,D:0.3%],F:0.8%,M:2.4%,n:5991

*BUSCO scores based on the hymenoptera_odb10 BUSCO set using v5.1.2. C= complete [S= single copy, D=duplicated], F=fragmented, M=missing, n=number of orthologues in comparison. A full set of BUSCO scores is available at https://blobtoolkit.genomehubs.org/view/iyCerRyby1.1/dataset/CAJUXP01/busco.

**Figure 3.**
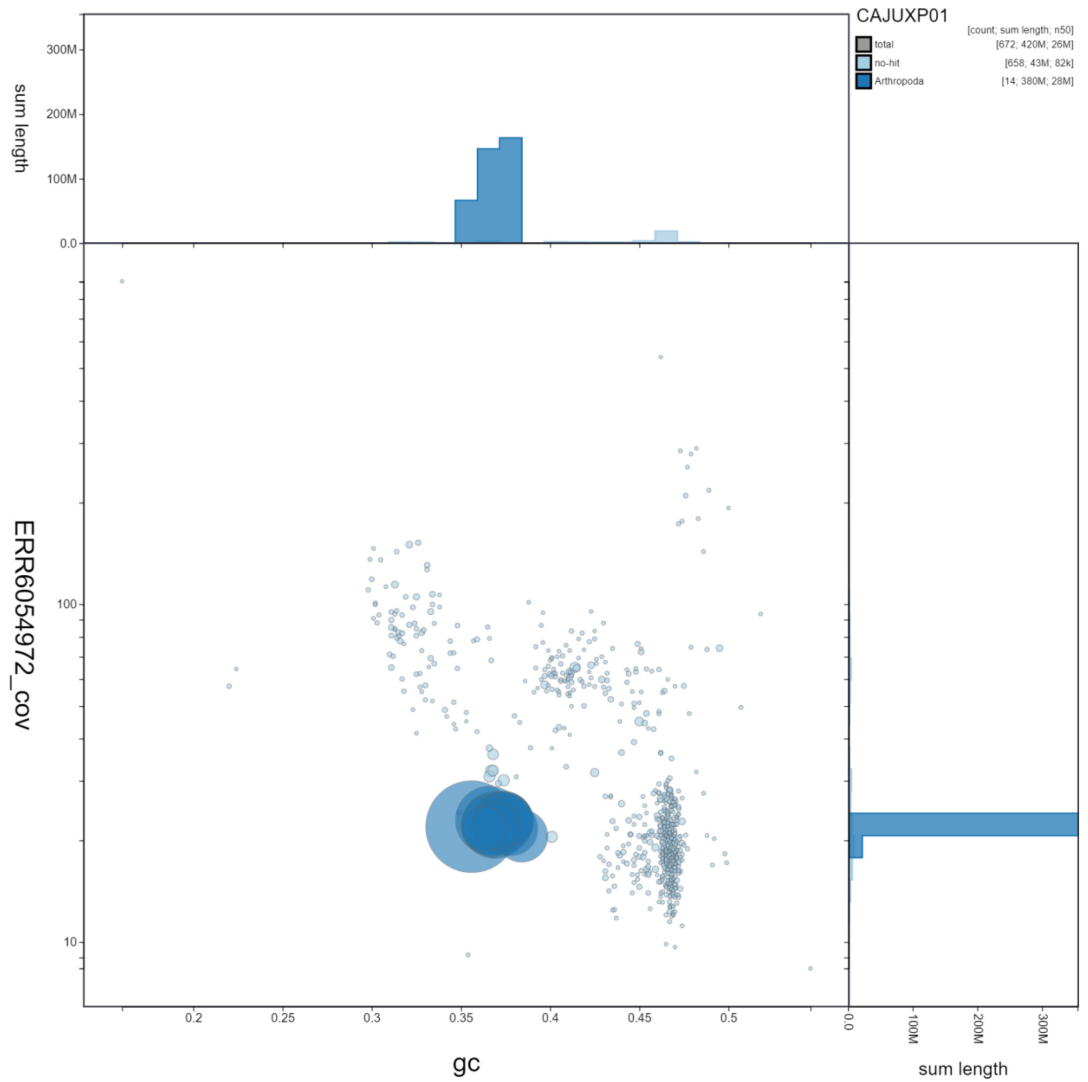
Genome assembly of *Cerceris rybyensis*, iyCerRyby1.1: GC coverage. BlobToolKit GC-coverage plot. Scaffolds are coloured by phylum. Circles are sized in proportion to scaffold length. Histograms show the distribution of scaffold length sum along each axis. An interactive version of this figure is available at https://blobtoolkit.genomehubs.org/view/iyCerRyby1.1/dataset/CAJUXP01/blob.

**Figure 4.**
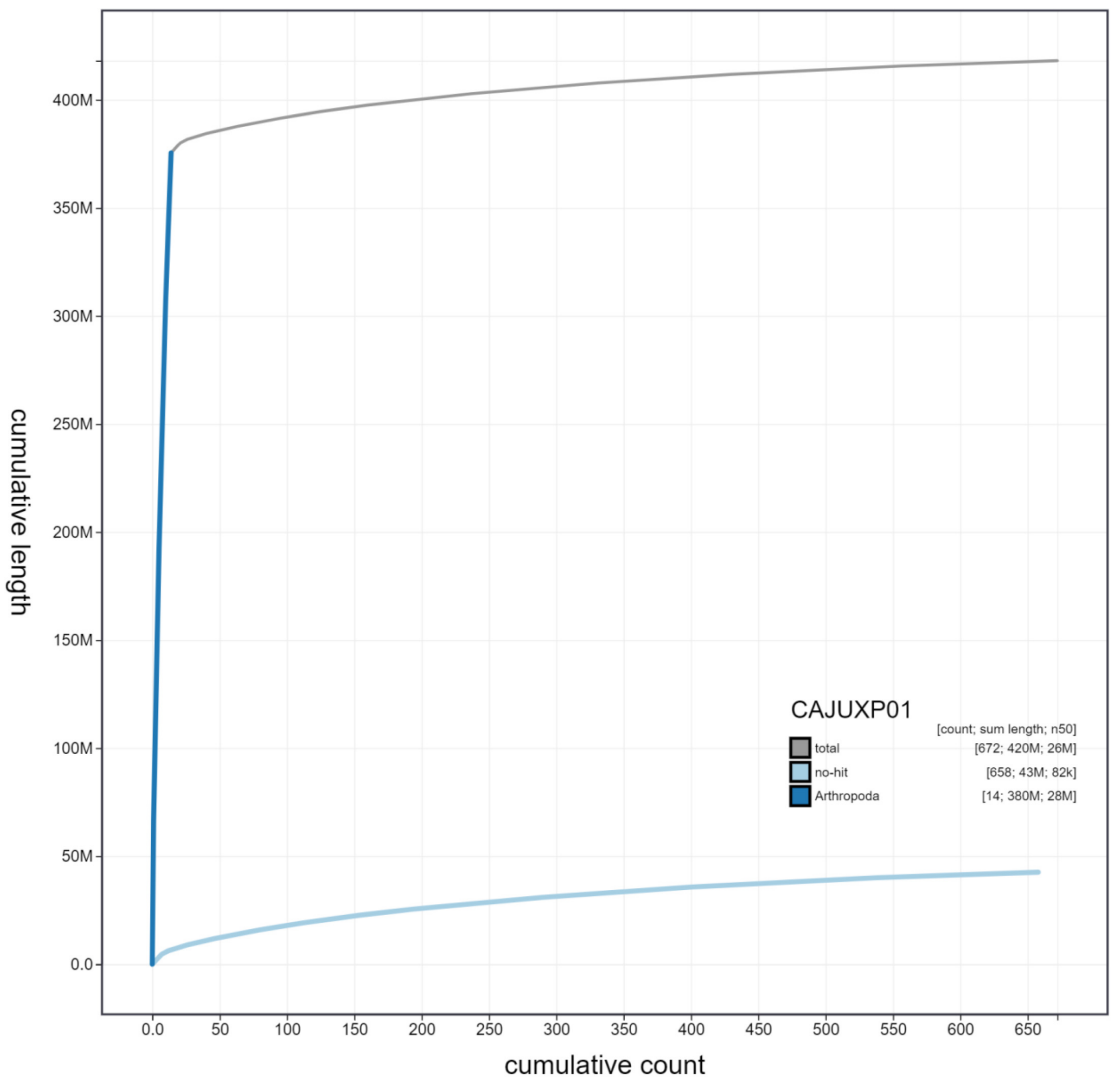
Genome assembly of *Cerceris rybyensis*, iyCerRyby1.1: cumulative sequence. BlobToolKit cumulative sequence plot. The grey line shows cumulative length for all scaffolds. Coloured lines show cumulative lengths of scaffolds assigned to each phylum using the buscogenes taxrule. An interactive version of this figure is available at https://blobtoolkit.genomehubs.org/view/iyCerRyby1.1/dataset/CAJUXP01/cumulative.

**Figure 5.**
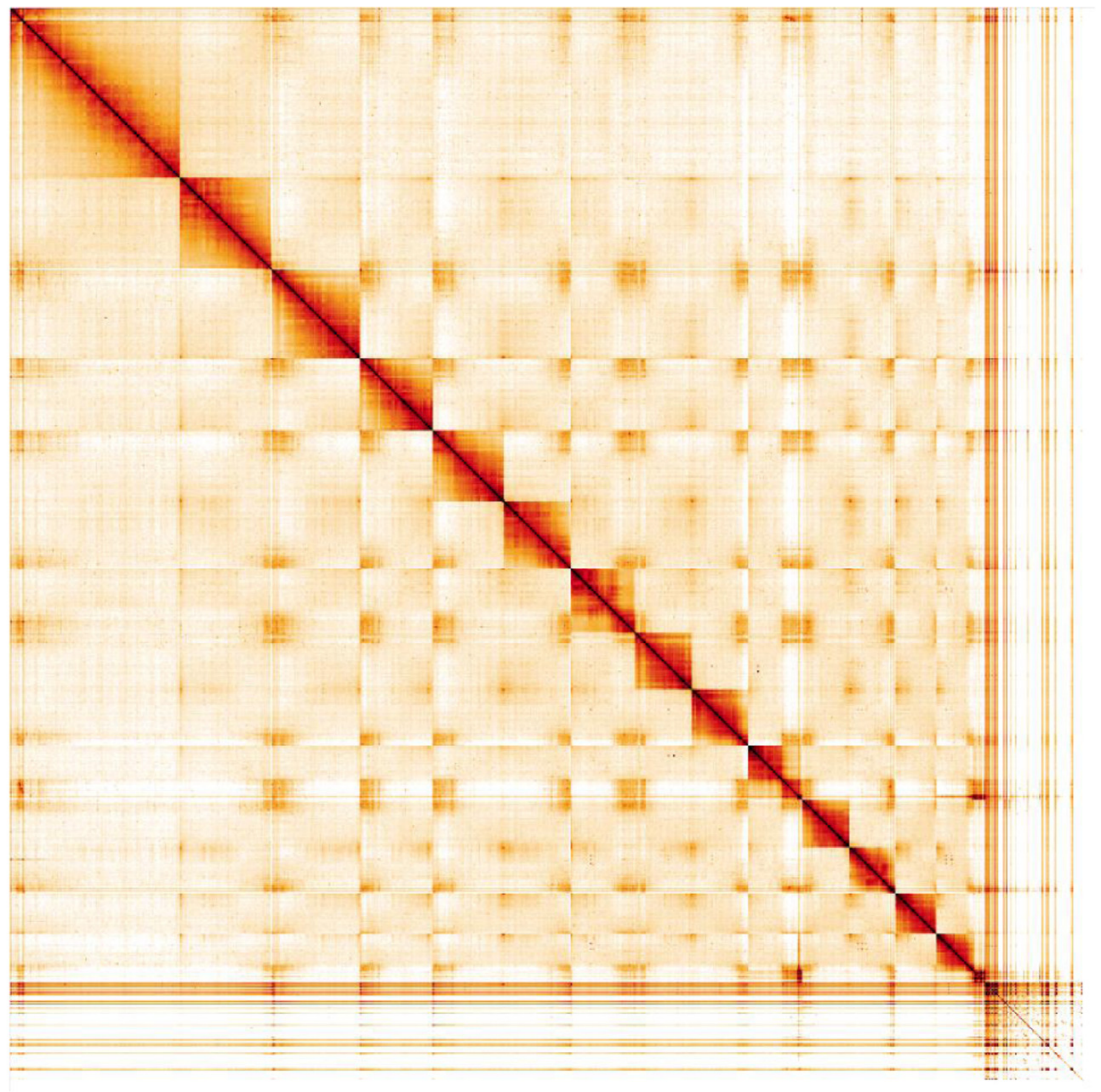
Genome assembly of *Cerceris rybyensis*, iyCerRyby1.1: Hi-C contact map. Hi-C contact map of the iyCerRyby1.1 assembly, visualised in HiGlass. Chromosomes are shown in order of size from left to right and top to bottom, with unassigned scaffolds shown in the bottom right.

**Table 2.** Chromosomal pseudomolecules in the genome assembly of *Cerceris rybyensis*, iyCerRyby1.1.

INSDC accession	Chromosome	Size (Mb)	GC%
OU342789.1	1	66.38	35.6
OU342790.1	2	35.56	36.6
OU342791.1	3	34.31	36.9
OU342792.1	4	28.31	37.3
OU342793.1	5	27.62	36.7
OU342794.1	6	26.18	37.4
OU342795.1	7	25.06	37.4
OU342796.1	8	22.18	37.5
OU342797.1	9	21.96	37.8
OU342798.1	10	20.77	38.4
OU342799.1	11	18.65	37.7
OU342800.1	12	17.68	36.8
OU342801.1	13	16.24	37.0
OU342802.1	14	14.61	36.3
OU342803.1	MT	0.02	16.3
-	Unplaced	42.57	43.0

## Methods

### Sample acquisition and DNA extraction

A single female *C. rybyensis* was collected from Wytham Woods, Oxfordshire, UK (latitude 51.772, longitude -1.338) by Liam Crowley, University of Oxford, using a net. The specimen was identified by the same individual and preserved on dry ice prior to transfer to the Wellcome Sanger Institute.

DNA was extracted at the Tree of Life laboratory, Wellcome Sanger Institute. The iyCerRyby1 sample was weighed and dissected on dry ice with tissue set aside for Hi-C sequencing. Abdomen tissue was disrupted using a Nippi Powermasher fitted with a BioMasher pestle. Fragment size analysis of 0.01–0.5 ng of DNA was then performed using an Agilent FemtoPulse. High molecular weight (HMW) DNA was extracted using the Qiagen MagAttract HMW DNA extraction kit. Low molecular weight DNA was removed from a 200-ng aliquot of extracted DNA using 0.8X AMpure XP purification kit prior to 10X Chromium sequencing; a minimum of 50 ng DNA was submitted for 10X sequencing. HMW DNA was sheared into an average fragment size between 12–20 kb in a Megaruptor 3 system with speed setting 30. Sheared DNA was purified by solid-phase reversible immobilisation using AMPure PB beads with a 1.8X ratio of beads to sample to remove the shorter fragments and concentrate the DNA sample. The concentration of the sheared and purified DNA was assessed using a Nanodrop spectrophotometer and Qubit Fluorometer and Qubit dsDNA High Sensitivity Assay kit. Fragment size distribution was evaluated by running the sample on the FemtoPulse system.

### Sequencing

Pacific Biosciences HiFi circular consensus and 10X Genomics read cloud sequencing libraries were constructed according to the manufacturers’ instructions. Sequencing was performed by the Scientific Operations core at the Wellcome Sanger Institute on Pacific Biosciences SEQUEL II and Illumina NovaSeq 6000 instruments. Hi-C data were generated from remaining thorax/abdomen tissue using the Arima Hi-C+ kit and sequenced on a NovaSeq 6000 instrument.

### Genome assembly

Assembly was carried out with Hifiasm ( Cheng *et al.*, 2021); haplotypic duplication was identified and removed with purge_dups ( Guan *et al.*, 2020). One round of polishing was performed by aligning 10X Genomics read data to the assembly with longranger align, calling variants with freebayes ( Garrison & Marth, 2012). The assembly was then scaffolded with Hi-C data ( Rao *et al.*, 2014) using SALSA2 ( Ghurye *et al.*, 2019). The assembly was checked for contamination and corrected using the gEVAL system ( Chow *et al.*, 2016) as described previously ( Howe *et al.*, 2021). Manual curation ( Howe *et al.*, 2021) was performed using gEVAL, HiGlass ( Kerpedjiev *et al.*, 2018) and Pretext. The mitochondrial genome was assembled using MitoHiFi ( Uliano-Silva *et al.*, 2021) and annotated using MitoFinder ( Allio *et al.*, 2020). The genome was analysed and BUSCO scores generated within the BlobToolKit environment ( Challis *et al.*, 2020). Table 3 contains a list of all software tool versions used, where appropriate.

**Table 3.** Software tools used.

Software tool	Version	Source
Hifiasm	0.15.1	Cheng *et al.*, 2021
purge_dups	1.2.3	Guan *et al.*, 2020
SALSA2	2.2	Ghurye *et al.*, 2019
longranger align	2.2.2	https://support.10xgenomics.com/genome-exome/software/pipelines/latest/advanced/other-pipelines
freebayes	1.3.1-17-gaa2ace8	Garrison & Marth, 2012
MitoHiFi	2	Uliano-Silva *et al.*, 2021
gEVAL	N/A	Chow *et al.*, 2016
HiGlass	1.11.6	Kerpedjiev *et al.*, 2018
PretextView	0.2.x	https://github.com/wtsi-hpag/PretextView
BlobToolKit	2.6.2	Challis *et al.*, 2020

### Ethics/compliance issues

The materials that have contributed to this genome note have been supplied by a Darwin Tree of Life Partner. The submission of materials by a Darwin Tree of Life Partner is subject to the Darwin Tree of Life Project Sampling Code of Practice. By agreeing with and signing up to the Sampling Code of Practice, the Darwin Tree of Life Partner agrees they will meet the legal and ethical requirements and standards set out within this document in respect of all samples acquired for, and supplied to, the Darwin Tree of Life Project. Each transfer of samples is further undertaken according to a Research Collaboration Agreement or Material Transfer Agreement entered into by the Darwin Tree of Life Partner, Genome Research Limited (operating as the Wellcome Sanger Institute), and in some circumstances other Darwin Tree of Life collaborators.

## Data availability

European Nucleotide Archive: Cerceris rybyensis (ornate tailed digger wasp). Accession number PRJEB45199; https://www.ebi.ac.uk/ena/browser/view/PRJEB45199.

The genome sequence is released openly for reuse. The *C. rybyensis* genome sequencing initiative is part of the Darwin Tree of Life (DToL) project. All raw sequence data and the assembly have been deposited in INSDC databases. The genome will be annotated and presented through the Ensembl pipeline at the European Bioinformatics Institute. Raw data and assembly accession identifiers are reported in Table 1.

## Author information

Members of the University of Oxford and Wytham Woods Genome Acquisition Lab are listed here: https://doi.org/10.5281/zenodo.5746938.

Members of the Darwin Tree of Life Barcoding collective are listed here: https://doi.org/10.5281/zenodo.5744972.

Members of the Wellcome Sanger Institute Tree of Life programme are listed here: https://doi.org/10.5281/zenodo.5744840.

Members of Wellcome Sanger Institute Scientific Operations: DNA Pipelines collective are listed here: https://doi.org/10.5281/zenodo.5746904.

Members of the Tree of Life Core Informatics collective are listed here: https://doi.org/10.5281/zenodo.5743293.

Members of the Darwin Tree of Life Consortium are listed here: https://doi.org/10.5281/zenodo.5638618.
