# Supplementary material for: The genome sequence of the ornate tailed digger wasp, Cerceris rybyensis (Linnaeus, 1771)
Source: Wellcome Open Res. 2021 Dec 13;6:337. [Version 1] doi: 10.12688/wellcomeopenres.17483.1 (PMC8980675; doi:10.12688/wellcomeopenres.17483.1)
Supplement: Peer review history

# Peer review: Reviewer response for version 1

**DOI:** 10.21956/wellcomeopenres.19333.r49292

**Published:** 2022-04-04

**Authors:** Rodolpho S. T. Menezes

**Affiliations:** 1 Universidade Federal de Santa Maria, Santa Maria, RS, Brazil

This short genome report describes the genome sequencing of the ornate tailed digger wasp, *Cerceris rybyensis*. The work begins with an introduction to the natural history of the wasp and describes the collection, DNA extraction, sequencing, and assembly. The manuscript is well written. I have only a few minor suggestions:

 Abstract:

- Please correct the species taxonomy information.

Background:

- “Genus”; “genus”

Sequencing:

- Why did you use thorax/abdomen tissue and not thorax and legs tissues?

Genome assembly:

- Since you assembled the mitochondrial genome, I think it is interesting that you show general (size, GC/AT content…) mitochondrial information about this species.

Are sufficient details of methods and materials provided to allow replication by others?

Yes

Is the rationale for creating the dataset(s) clearly described?

Yes

Are the datasets clearly presented in a useable and accessible format?

Yes

Are the protocols appropriate and is the work technically sound?

Yes

Reviewer Expertise:

Phylogenomics, Phylogeography, and Cytogenetics.

I confirm that I have read this submission and believe that I have an appropriate level of expertise to confirm that it is of an acceptable scientific standard.

# Peer review: Reviewer response for version 1

**DOI:** 10.21956/wellcomeopenres.19333.r47602

**Published:** 2022-01-26

**Authors:** Peter Dearden

**Affiliations:** 1 Genomics Aotearoa, Department of Biochemistry, University of Otago, Dunedin, New Zealand

This manuscript describes the sequencing of the ornate-tailed digger wasp. The work describes the collection, DNA extraction sequencing, assembly, and analysis of the genome, providing all of the necessary metrics. The manuscript is well written, and the figures clear and well explained. Methods are detailed and easy to follow. The sequence produced will be a helpful addition to hymenopteran genomics and the detailed and careful description of the work make this a useful resource for future work.

 This work is scientifically sound and I recommend its approval.

Are sufficient details of methods and materials provided to allow replication by others?

Yes

Is the rationale for creating the dataset(s) clearly described?

Yes

Are the datasets clearly presented in a useable and accessible format?

Yes

Are the protocols appropriate and is the work technically sound?

Yes

Reviewer Expertise:

Genomics

I confirm that I have read this submission and believe that I have an appropriate level of expertise to confirm that it is of an acceptable scientific standard.
